# Nomogram based on spectral CT quantitative parameters and typical radiological features for distinguishing benign from malignant thyroid micro-nodules

**DOI:** 10.1186/s40644-023-00525-2

**Published:** 2023-01-26

**Authors:** Zuhua Song, Qian Li, Dan Zhang, Xiaojiao Li, Jiayi Yu, Qian Liu, Zongwen Li, Jie Huang, Xiaodi Zhang, Zhuoyue Tang

**Affiliations:** 1 Department of Radiology, Chongqing General Hospital, No.118, Xingguang Avenue, Liangjiang New Area, Chongqing, 401147 China; 2 Philips Healthcare, Chengdu branch, Chengdu, China

**Keywords:** Thyroid nodule, Spectral computed tomography, Nomogram, Diagnosis, Differential

## Abstract

**Purpose:**

To analyse the predictive effect of a nomogram combining dual-layer spectral computed tomography (DSCT) quantitative parameters with typical radiological features in distinguishing benign micro-nodule from thyroid microcarcinoma (TMC).

**Methods:**

Data from 342 instances with thyroid micro-nodules (≤1 cm) who underwent DSCT (benign group: *n* = 170; malignant group: *n* = 172) were reviewed. Typical radiological features including micro-calcification and enhanced blurring, and DSCT quantitative parameters including attenuation on virtual monoenergetic images (40 keV, 70 keV and 100 keV), the slope of the spectral HU curve (λHU), normalized iodine concentration (NIC), and normalized effective atomic number (NZeff) in the arterial phase (AP) and venous phase (VP), were measured and compared between the benign and malignant groups. The receiver operating characteristic (ROC) curve was used to assess the diagnostic performance of significant quantitative DSCT parameters or the models combining DSCT parameters respectively and typical radiological features based on multivariate logistic regression (LR) analysis. A nomogram was developed using predictors with the highest diagnostic performance in the above model, as determined by multivariate LR analysis.

**Results:**

The DSCT parameter APλHU showed the greatest diagnostic efficiency in identifying patients with TMC, with an area under the ROC curve (AUC) of 0.829, a sensitivity and specificity of 0.738 and 0.753, respectively. Then, APλHU was combined with the two radiological features to construct the DSCT-Radiological nomogram, which had an AUC of 0.858, a sensitivity of 0.791 and a specificity of 0.800. The calibration curve of the nomogram demonstrated that the prediction result was in good agreement with the actual observation. The decision curve revealed that the nomogram can result in a greater net benefit than the all/none-intervention strategy for all threshold probabilities.

**Conclusion:**

As a valid and visual noninvasive prediction tool, the DSCT-Radiological nomogram incorporating DSCT quantitative parameters and radiological features shows favourable predictive efficiency for identifying benign and malignant thyroid micro-nodules.

**Supplementary Information:**

The online version contains supplementary material available at 10.1186/s40644-023-00525-2.

## Introduction

Thyroid carcinoma is the most prevalent endocrine malignancy worldwide [1, 2]. Among the various types of thyroid carcinoma, thyroid microcarcinoma (TMC) refers to nodules with a maximum diameter of less than 1 cm according to the World Health Organization guidelines [3, 4]. According to recent research, the detection rate of TMC is quickly increasing globally largely due to improved diagnostic methods, which has attracted extensive attention from many scholars [5, 6]. However, high-risk TMC which have evidence of extrathyroidal extension, lymph nodes or distant metastasis should be performed surgical resection. Benign thyroid micro-nodules are generally treated by ensuring patient comfort and performing follow-up examinations rather than by surgery [7]. In addition, there are several different follow-up strategies for malignant and benign micronodules [8]. Thus, proper patient selection will resolve the troublesome issue of overtreatment and avoid unnecessary surgical intervention which can lead to surgical complications such as vocal cord paralysis and permanent hypothyroidism.

Several different procedures are used to diagnose a thyroid micro-nodule, including physical examination, blood test, ultrasound (US), computed tomography (CT) and biopsy. US is the first-line imaging modality for the detection and risk stratification of thyroid nodules [9]. However, misdiagnosis is still prevalent for certain thyroid micro-nodules because of the subjective judgment differences between operators, similarities in some ultrasound features between benign and malignant micro-nodules, and various diagnostic standards in different geographic areas [10, 11]. US-guided fine-needle aspiration biopsy (FNAB) is currently the most reliable and cost-effective method for evaluating thyroid nodules [8], However, the American Thyroid Association guidelines do not recommend FNAB for evaluating thyroid nodules less than or equal to 1 cm [12]. Although the noninvasive detection of thyroid micro-nodules has become easier in recent years due to rapid development in US and CT, the precise identification of TMC and benign micro-nodule still faces certain challenges.

Dual-layer spectral CT (DSCT) is a novel energy imaging modality that offers multiparametric data not obtained by conventional CT imaging, including iodine concentration (IC) and effective atomic number (Zeff) [13]. This method also has several advantages, such as increased sensitivity and qualitative accuracy of lesion detection, the ability to determine material composition and minimization of metallic artefacts [14]. Previous studies have revealed that some energy-related quantitative CT parameters, especially the IC value, have potential diagnostic value for the differentiation of benign and malignant thyroid nodules [15–17]. However, the association between multiple DSCT-based quantitative parameters and the precise identification of TMC and benign micro-nodule is still unclear and is not frequently reported.

In this study, we hypothesized that multiple DSCT quantitative parameters of thyroid micro-nodule may help to distinguish TMC patients. To test our hypothesis, we sought to establish a DSCT-Radiological nomogram combining the quantitative energy parameters and typical radiological features to identify TMC patients to aid in individual clinical decision-making.

## Materials and methods

### Patient selection

This retrospective study was approved by the medical ethics committee of the Chongqing General Hospital and the requirement for written informed consent was waived. From December 2019 to November 2021, 249 patients with thyroid micro-nodules (≤1 cm) who received DSCT scans for preoperative assessment and pathological diagnosis were recruited for our study. The exclusion criteria were as follows: (1) patients who had received any therapy before DSCT examination (*n* = 12); (2) the image quality was not sufficient for measurement due to obvious artefacts or noise (*n* = 3); (3) cases where the thyroid micro-nodules were invisible or were not clearly distinguished on DSCT images(*n* = 5); (4) patients who had received any biopsy before the DSCT examination (*n* = 9); (5) patients with diffuse thyroid disease (*n* = 12).

Ultimately, 342 thyroid micro-nodules (172 malignant micro-nodules and 170 benign micro-nodules) from 208 patients were included.

### DSCT image acquisition

The participants in our study underwent unenhanced and contrast-enhanced neck examination though a 64-slice DSCT devices (IQon Spectral CT, Philips Healthcare, Amsterdam, The Netherlands) using the following scan protocol: tube voltage, 120 kV; tube current, modulated with automated exposure control (DoseRight system, Philips Healthcare); detector collimation, 64 × 0.625 mm; field of view, 350 mm; matrix, 512 × 512; layer thickness, 5 mm; reconstruction thickness, 1.25 mm. Nonionic contrast media (Iohexol, 350 mgI/ml, Schering, Berlin, Germany) was injected though an automatic injector at a dose of 1.5 ml/kg and the injection rate 3.5 ml/s, followed by 30 ml of saline flashing at the same rate. After non-contrast CT scanning, contrast-enhanced CT scanning was conducted. The trigger point was set in the descending aorta lumen at the tracheal bifurcation level. The trigger threshold was 150 Hounsfield units (HUs). The venous phase (VP) started to be collected 40 seconds after the end of the arterial phase (AP) scan.

### DSCT parameter analysis

All images of the AP and VP phases were transferred to a spectral CT dedicated post-processing workstation (IntelliSpace Portal Version 10.1, Philips Healthcare, Amsterdam, The Netherlands) for subsequent quantitative analysis. The detailed steps are as follows: ① Virtual monoenergetic maps, iodine-based material decomposition map and effective atomic number map were reconstructed for each DSCT scan based on raw imaging data. ② A circular ROI (range 2.35–52.51 mm^2^) was manually drawn as large as possible to cover no less than 2/3 of the area of the thyroid micro-nodule on the cross sectional map, avoiding obvious necrotic or cystic areas and calcification. The ROI was placed on the core area of the carotid artery at the same level. The location, shape and size of ROIs were kept constant at the same level in different phases using the copy-and-paste function. ③ To reduce the measurement variation, two sets of measurements of all DSCT parameters were obtained independently by two radiologists each with 6 and 14 years of experience, after which the average values of the 2 measurements were used as the final results for subsequent analyses. Inter-reader agreement was assessed to evaluate the DSCT parameters by intraclass correlation coefficient (ICC), with details described in the Supplementary Material (Table S1).

The following measured parameters were automatically calculated and used in our study: (I) CT value (HU) from 40 keV, 70 keV and 100 keV monoenergetic maps in the AP and VP phase were measured: AP_40keV_, AP_70keV_, AP_100keV_, VP_40keV_, VP_70keV_ and VP_100keV_; (II) The slope of spectral HU curve (λHU) in the AP and VP phase: λHU = (CT value at 40 keV – CT value at 100 keV) / (100–40) (the changes in the CT attenuation in this energy levels showed larger changes and differences in contrast to the other energy levels); (III) The iodine concentration (IC) and effective atomic number (Zeff) were measured on the iodine-based material decomposition maps and effective atomic number maps; (IV) APNIC = IC of lesion / IC of the carotid artery, APNZeff = Zeff of lesion / Zeff of the carotid artery, VPNIC = IC of lesion / IC of the carotid artery, VPNZeff = Zeff of lesion / Zeff of the carotid artery.

### Image sign analysis

Qualitative analysis of the typical radiological features of thyroid micro-nodules was performed by two radiologists completed fellowship trained in head and neck imaging with 8 and 17 years of experience of post-training and were blinded to the pathological results to mitigate potential cognitive biases based on the non-contrast, AP and VP images. Any disagreements were resolved by consensus. The following radiological features were analyzed: micro-calcification (the diameter ≤ 2 mm) and enhanced blurring (the nodule-thyroid junction was more obscure and the density difference between the nodules and the thyroid was smaller after contrast enhancement).

### Statistical analysis

All statistical analyses were performed using R software (http://www.R-project.org) and SPSS software (version 25.0, SPSS, IBM). The typical radiological features and DSCT parameters were assessed by applying a two-sample t test or the Mann-Whitney U test, and chi-squared test, where appropriate. A two-sided *P* value < 0.05 indicated statistical significance. The diagnostic performance of significant quantitative DSCT parameters, or the models combining DSCT parameters respectively and typical radiological features based on multivariate logistic regression (LR) analysis, was assessed using the receiver operating characteristic (ROC) curves. The area under the ROC curve (AUC), with expressing 95% confidence intervals (95% CIs), was obtained to compare the diagnostic capability. After the cut-off value had been determined, the sensitivity and specificity were calculated. The DeLong test was used to compare the AUCs among different combinations in our study. In addition, a nomogram based on the above model with the highest diagnostic performance was constructed to provide an easy-to-use and effective tool for clinicians to predict the probability of TMC in patients. Moreover, the fit goodness of the nomogram was evaluated using the calibration curve, while the clinical utility of the nomogram was determined using decision curve analysis (DCA).

## Results

### Demographics and thyroid nodules characteristics

Ultimately, a total of 342 thyroid micro-nodules in 208 patients, including 170 benign and 172 malignant micro-nodules. Among the patients, 85 were men (mean age ± standard deviation[SD], 43.29 ± 11.88 years) with an age range of 19–75 years, and 123 were women (mean age ± SD, 43.94 ± 10.89 years) with an age range of 21–72 years. According to the pathological results, the 172 malignant micronodules included 165 papillary carcinoma, 5 follicular carcinoma and 2 medullary carcinoma. The 170 benign micronodules included 137 nodular goiters, 26 follicular adenomas, 4 adenomatous nodular goiters and 3 inflammatory nodules.

### Comparison of DSCT parameters and the typical radiological features

The DSCT parameters and the typical radiological features in the benign and malignant micro-nodule cohorts are summarized in Table 1. According to the univariate analysis, both typical radiological features, including micro-calcification and enhanced blurring, were significantly different between the two groups (*p* <  0.05). The values of AP_40keV_, AP_70keV_, AP_100keV_, APλHU, APNIC and APNZeff in the benign group were all higher than those in the malignant group (all *p* <  0.05). The mean values of VPNIC and NZeffVP were 0.68 ± 0.11 and 0.94 ± 0.02, respectively, in the benign group and were 0.72 ± 0.14 and 0.95 ± 0.03, respectively, in the malignant group, and the differences ware statistically significant (NICVP, *p* = 0.003; NZeffVP, *p* = 0.009). However, the other parameters of the VP between the two groups were no not significantly different (*p* > 0.05) (Table 1).

**Table 1:** Univariate analysis in the benign and malignant micro-nodule cohort

Variable	Benign micro-nodule cohort (*n* = 170)	malignant micro-nodule cohort (*n* = 172)	F value /Zvalue /X^2^value	*P* value
**AP**				
40 keV(HU)	360.34 ± 53.26	282.83 ± 62.87	3.915	< 0.001
70 keV(HU)	135.40 (123.98, 148.65)	114.60 (99.78, 127.45)	−8.514	< 0.001
100 keV(HU)	81.50 (70.40, 92.80)	75.05 (64.50, 84.96)	−3.814	< 0.001
λHU	4.67 ± 0.83	3.48 ± 0.93	1.780	< 0.001
NIC	0.37 (0.32, 0.42)	0.30 (0.23, 0.34)	−8.974	< 0.001
NZeff	0.82 (0.80, 0.85)	0.80 (0.77, 0.82)	−6.507	< 0.001
**VP**				
40 keV(HU)	281.95 (257.60, 317.55)	282.45 (246.02, 324.75)	−0.365	0.715
70 keV(HU)	117.50 (105.63, 128.28)	115.35 (103.10, 130.78)	−0.103	0.918
100 keV(HU)	74.06 ± 14.99	75.53 ± 13.04	1.867	0.334
λHU	3.50 (3.06, 4.03)	3.46 (2.92, 4.04)	−0.502	0.616
NIC	0.68 ± 0.11	0.72 ± 0.14	17.013	0.003
NZeff	0.94 ± 0.02	0.95 ± 0.03	12.280	0.009
**Radiological features**				
Micro-calcification (presence)	46 (27.1%)	89 (51.7%)	21.806	< 0.001
Enhanced blurring (presence)	62 (36.5%)	97 (56.4%)	13.644	< 0.001

*AP* arterial phase, *VP* venous phase, *HU* CT value, *λHU* the slope of spectral HU curve, *NIC* normalized iodine concentration, *NZeff* normalized effective atomic number

### Diagnostic effectiveness of DSCT parameters

The AUC, sensitivity and specificity of each DSCT quantitative parameter are shown in Table 2. the APλHU had the highest diagnostic performance than the other quantitative parameters (AUC = 0.829), with a sensitivity and specificity of 0.738 and 0.753, respectively.

**Table 2:** Diagnostic effectiveness of DSCT Parameters

DSCT Parameters	AUC(95CI%)	Sensitivity	Specificity	Cutoff value	*P* value
AP_40keV_	0.825 (0.781–0.869)	0.744	0.753	312.20	< 0.001
AP_70keV_	0.766 (0.716–0.817)	0.709	0.735	126.35	< 0.001
AP_100keV_	0.619 (0.560–0.679)	0.581	0.594	79.60	< 0.001
APλHU	0.829 (0.787–0.872)	0.738	0.753	3.89	< 0.001
APNIC	0.781 (0.732–0.829)	0.733	0.706	0.33	< 0.001
APNZeff	0.703 (0.649–0.758)	0.624	0.663	0.82	< 0.001
VPNIC	0.588 (0.527–0.648)	0.558	0.571	0.75	0.005
VPNZeff	0.606 (0.545–0.666)	0.547	0.605	0.96	< 0.001

*AP* arterial phase, *VP* venous phase, *λHU* the slope of spectral HU curve, *NIC* normalized iodine concentration, *NZeff* normalized effective atomic number

### Diagnostic value of the combination of DSCT parameters and typical radiological features

**Fig. 1:**
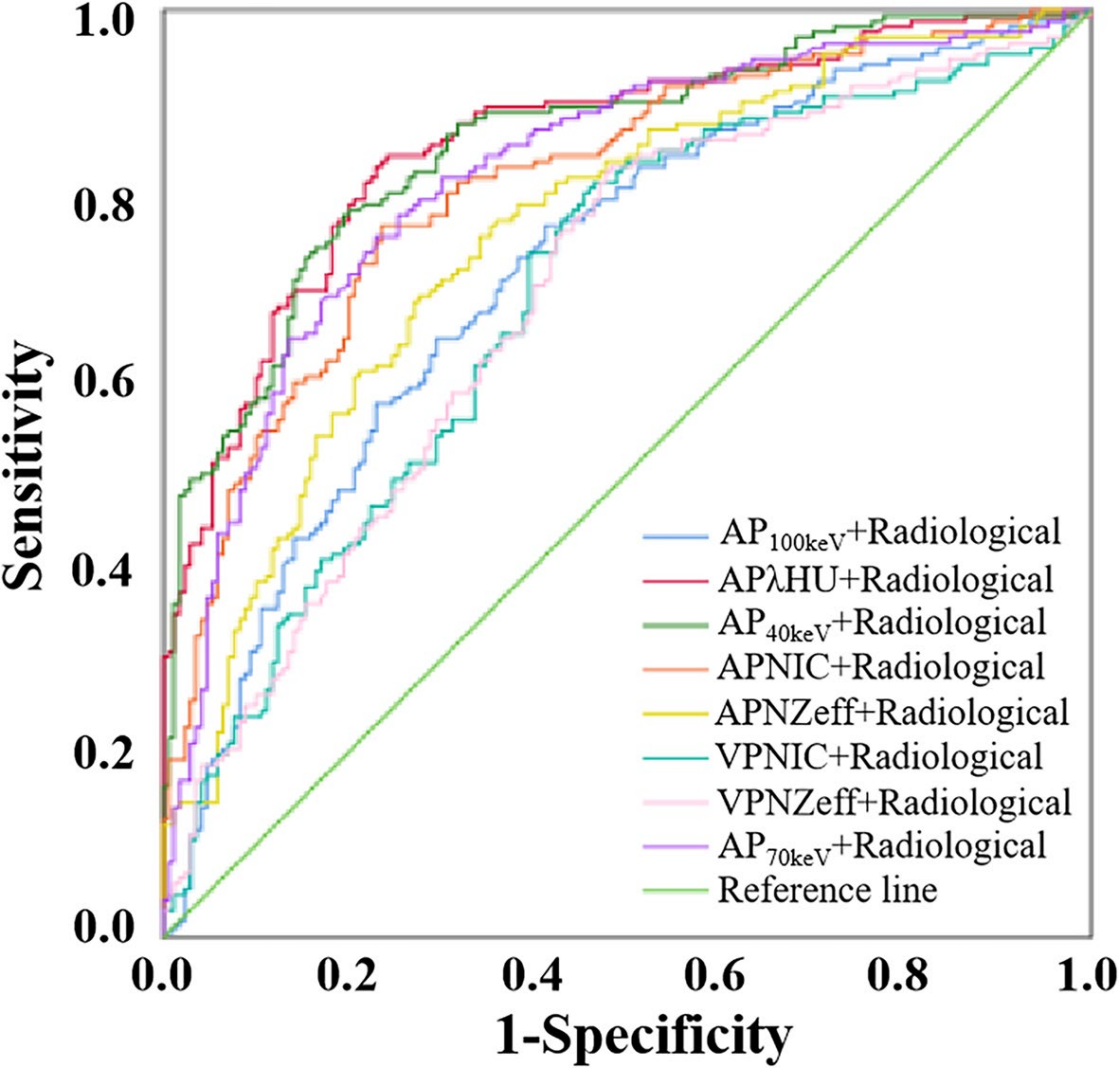
Receiver operating characteristic curves of the combined model with DSCT parameters and typical radiological features for identifying TMC and benign micro-nodule. *AP* arterial phase, *VP* venous phase, *HU* CT value, *λHU* the slope of spectral HU curve, *NIC* normalized iodine concentration, *NZeff* normalized effective atomic number

The combined models that incorporated the significant DSCT quantitative parameters respectively into the typical radiological features of micro-calcification and enhanced blurring were developed by multivariate LR analysis. The ROC curves of these combined models are shown in Fig. 1. Table 3 shows that the diagnostic performance of these combined models was better than that of the quantitative DSCT parameter alone. *P*-values of DeLong test for AUC of 8 different combinations are shown in Table S2. The APλHU+Radiological model, which combind APλHU with two typical radiological features, had the highest diagnostic performance with an AUC, sensitivity and specificity of 0.858, 0.791 and 0.800, respectively.

**Table 3:** Diagnostic performance of the combined model with DSCT parameters and typical radiological features

Combined model	AUC (95CI%)	Sensitivity	Specificity	Cutoff value	*P* value
AP_40keV_ + Radiological	0.857 (0.818–0.897)	0.767	0.806	−0.196	< 0.001
AP_70keV_ + Radiological	0.823 (0.778–0.867)	0.750	0.771	−0.014	< 0.001
AP_100keV_ + Radiological	0.721 (0.668–0.775)	0.641	0.680	−0.217	< 0.001
APλHU+Radiological	0.858 (0.819–0.898)	0.791	0.800	−0.069	< 0.001
APNIC+Radiological	0.813 (0.768–0.858)	0.729	0.767	0.026	< 0.001
APNZeff+Radiological	0.759 (0.709–0.810)	0.692	0.718	0.005	< 0.001
VPNIC+Radiological	0.693 (0.637–0.749)	0.640	0.635	−0.320	< 0.001
VPNZeff+Radiological	0.691 (0.635–0.747)	0.651	0.618	−0.396	< 0.001

*AP* arterial phase, *VP* venous phase, *λHU* the slope of spectral HU curve, *NIC* normalized iodine concentration, *NZeff* normalized effective atomic number

### Diagnostic effectiveness of the different combination of DSCT parameters APλHU or typical radiological features

**Fig. 2:**
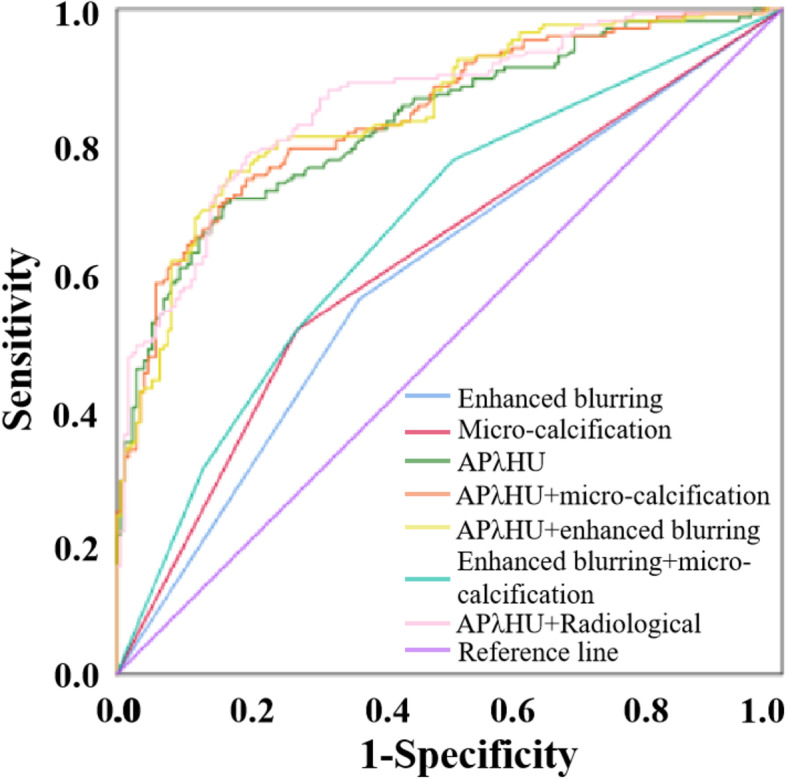
Receiver operating characteristic curves of the different combination of DSCT parameters APλHU or typical radiological features for identifying TMC and benign micro-nodule. *AP* arterial phase, *λHU* the slope of spectral HU curve

The ROC curves of these different combined models of APλHU and typical radiological features, APλHU or typical radiological features (micro-calcification and enhanced blurring) alone are shown in Fig. 2. Table 4 shows that the diagnostic performance of these different combined models, APλHU or typical radiological features alone. *P*-values of DeLong test for AUC of 7 different combinations are shown in Table S3.

**Table 4:** Diagnostic performance of the different combination of DSCT parameters APλHU or typical radiological features

Variable	AUC (95CI%)	Sensitivity	Specificity	Cutoff value	*P* value
Enhanced blurring	0.600 (0.540–0.660)	0.564	0.635	–	0.001
Micro-calcification	0.623 (0.564–0.683)	0.517	0.729	–	< 0.001
APλHU	0.829 (0.787–0.872)	0.738	0.753	3.89	< 0.001
Micro-calcification+enhanced blurring	0.668 (0.611–0.725)	0.773	0.494	–	< 0.001
APλHU+micro-calcification	0.842 (0.801–0.883)	0.771	0.762	0.11	< 0.001
APλHU+enhanced blurring	0.846 (0.806–0.887)	0.779	0.776	0.18	< 0.001
APλHU+Radiological	0.858 (0.819–0.898)	0.791	0.800	−0.07	< 0.001

*AP* arterial phase, *λHU* the slope of spectral HU curve

### Development and performance of the DSCT-radiological nomogram

**Fig. 3:**
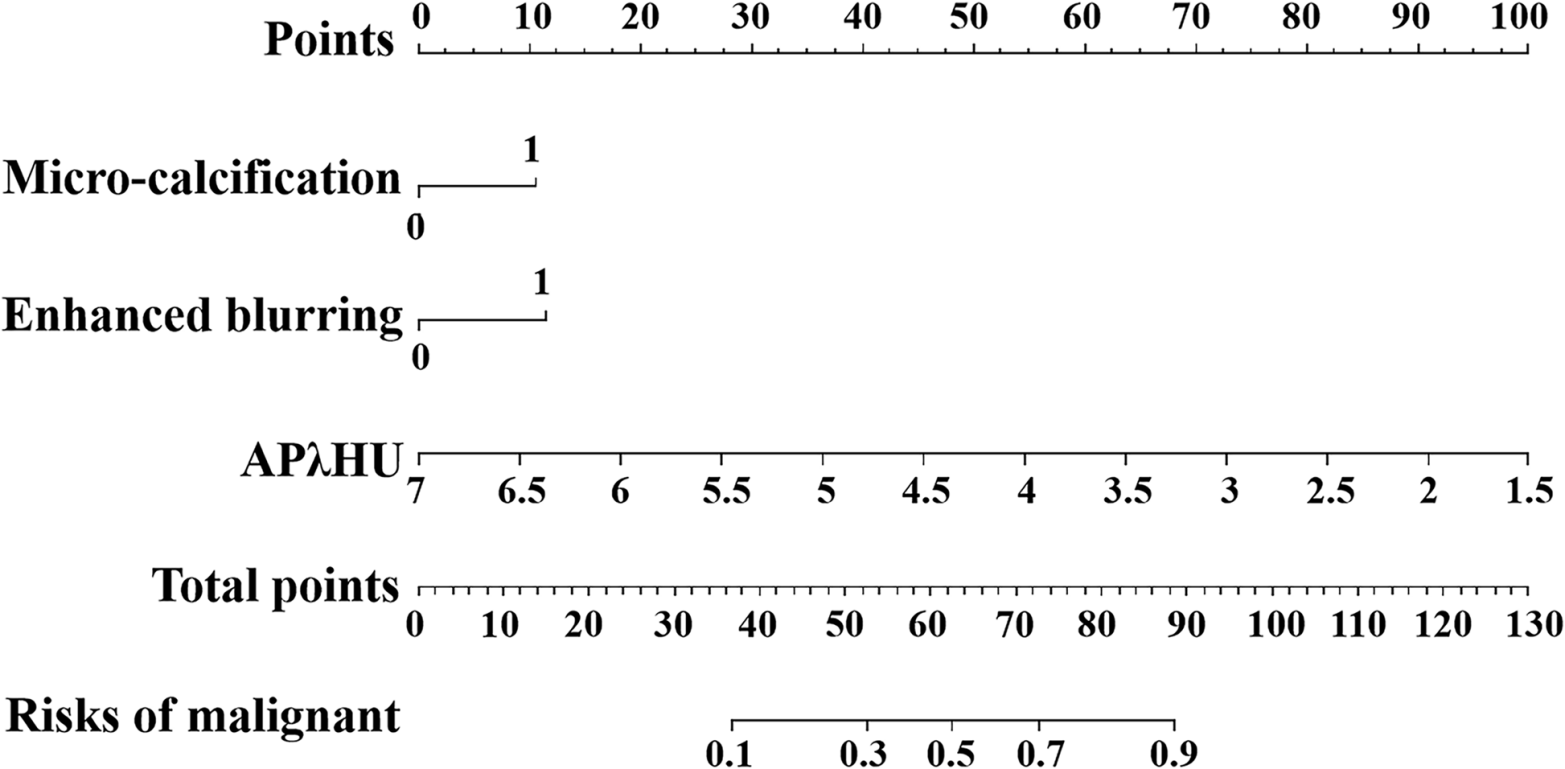
The DSCT-Radiological nomogram for assessing malignant micro-nodule risk. The method for calculating the risk of malignant micro-nodule was as follows. First, points for each variable are assigned by corresponding values from the “Points” axis. Second, the “Total points” is obtained by summing up points of all predictors. Third, a vertical line should be drawn down the total points to get the risk of malignant micro-nodule. *AP* arterial phase, *λHU* the slope of spectral HU curve

**Fig. 4:**
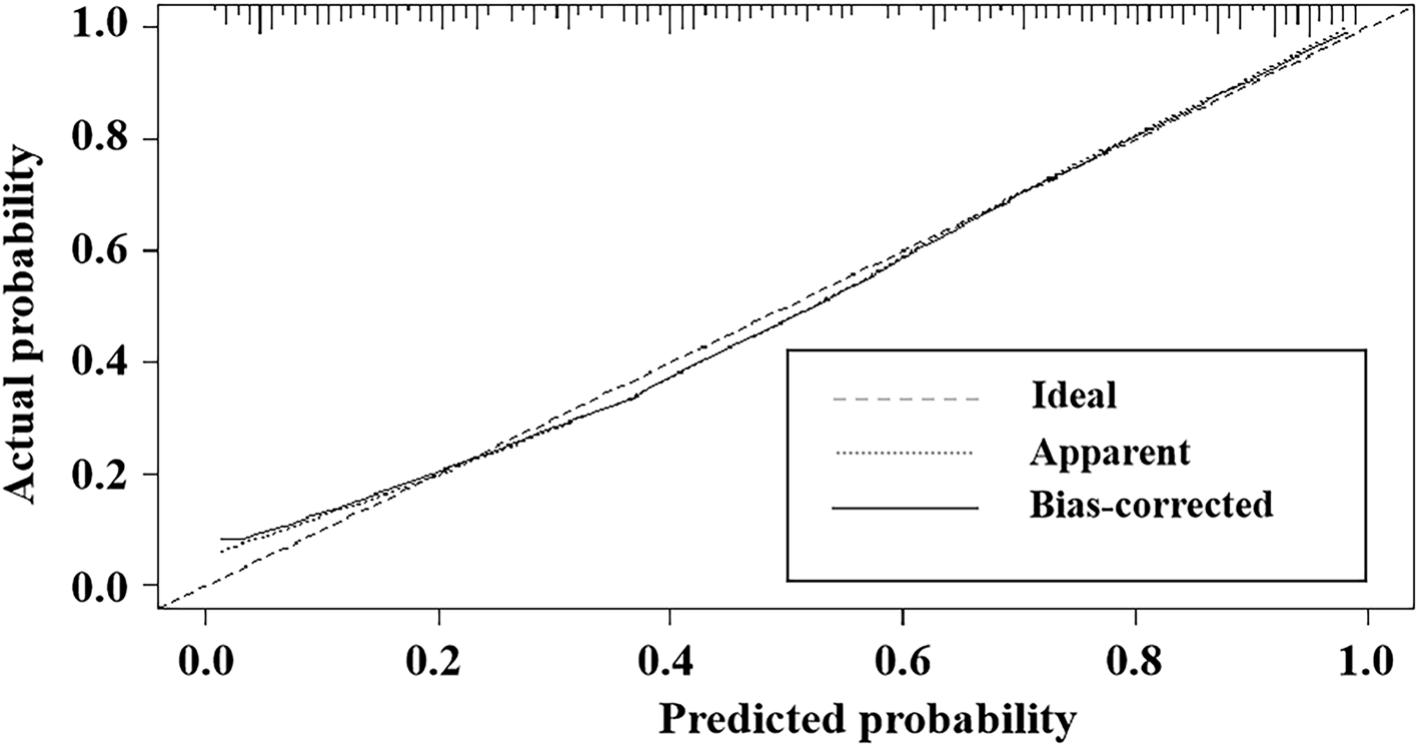
The Calibration curve for the DSCT-Radiological nomogram

**Fig. 5:**
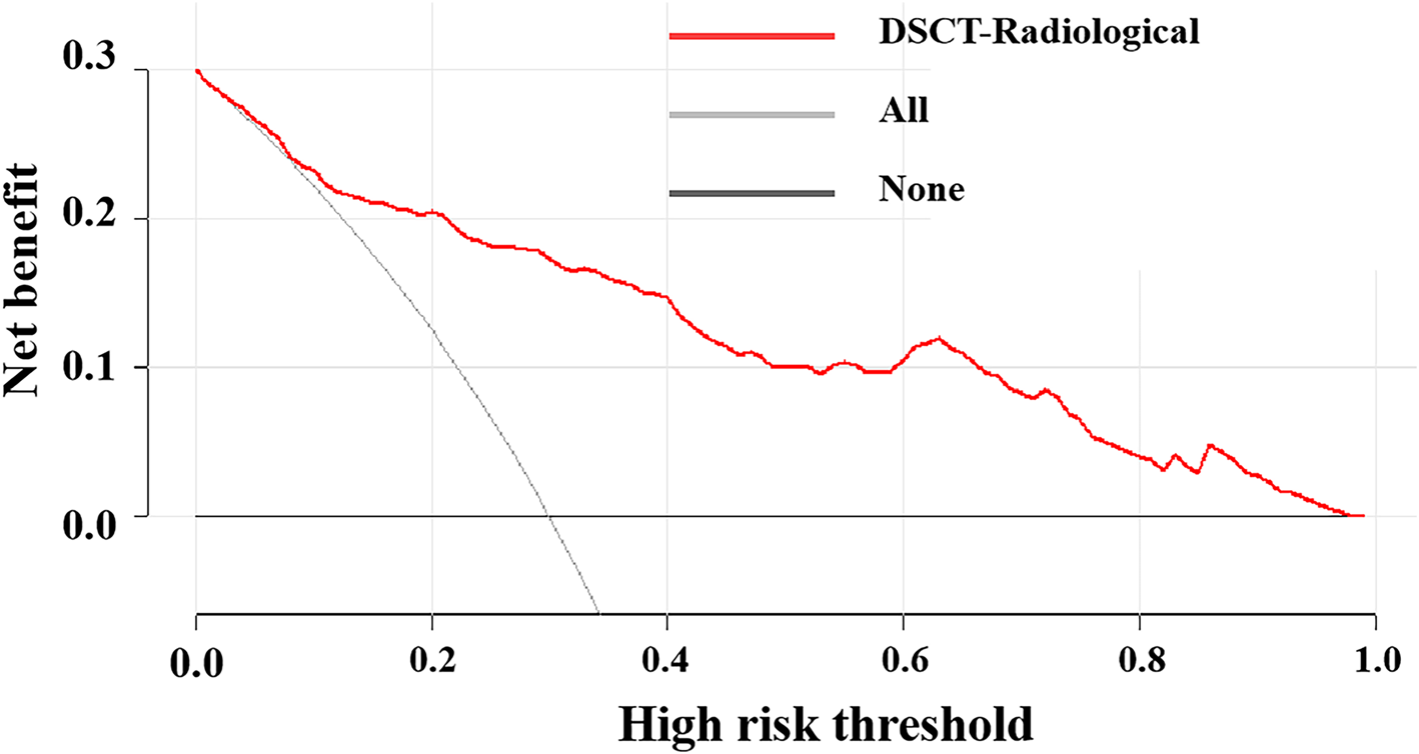
Decision curve analysis (DCA) of the DSCT-Radiological nomogram. The y-axis shows the net benefit. The x-axis shows the corresponding high risk threshold. The grey line represents the assumption that all micro-nodules were malignant. The black line represents the assumption that all micro-nodules were benign

**Fig. 6:**
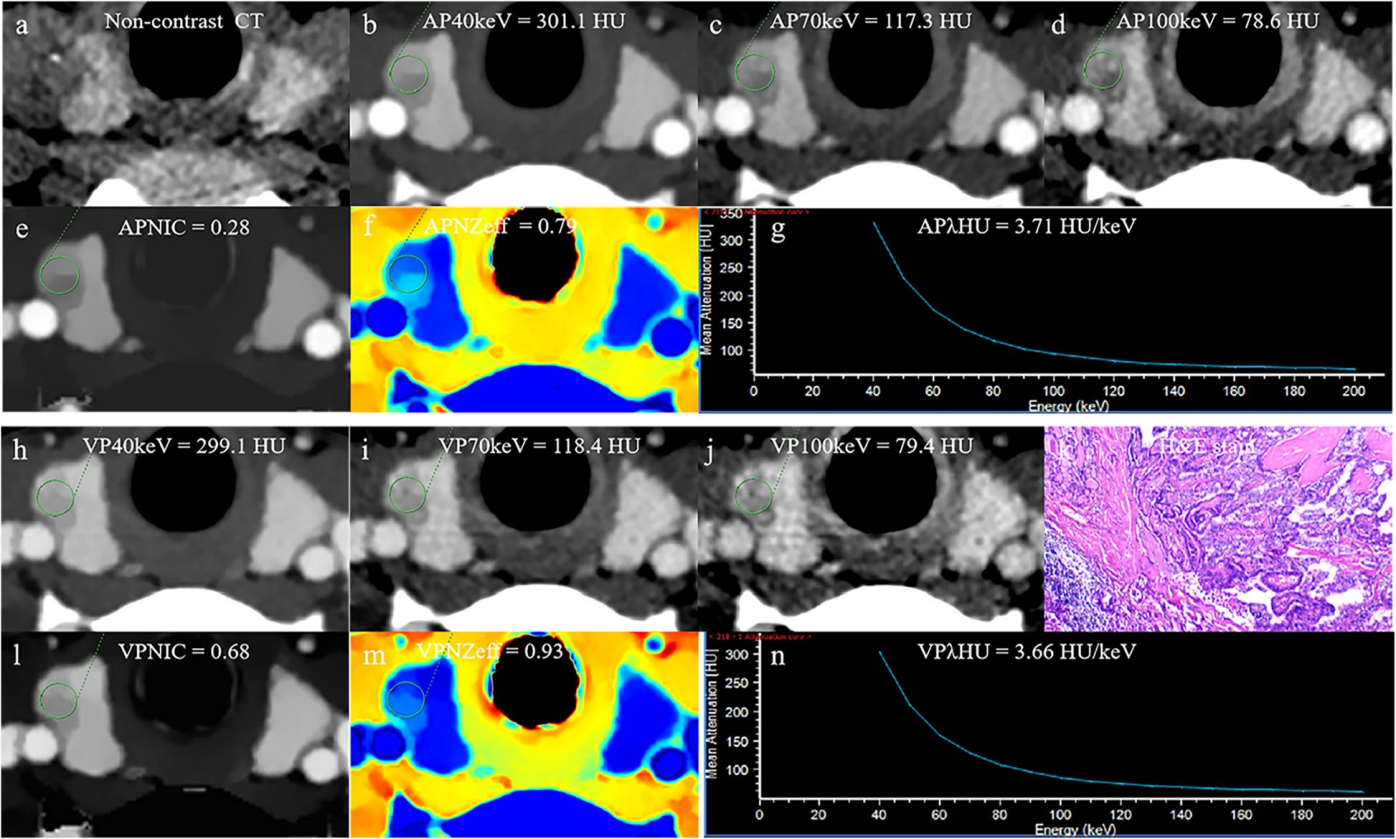
The DSCT quantitative parameters and Haematoxylin-eosin stain in a 30-year-old women with thyroid papillary carcinoma. **a** Axial non-contrast CT image shows micro-calcification in micro-nodule of thyroid right lobe. **b**-**d** CT value of arterial phase 40 keV, 70 keV and 100 keV monochromatic image is 301.1 HU, 117.3HU, 78.6HU, respectively. **e** Arterial phase NIC is 0.28. **f** Arterial phase NZeff is 0.79. **g** Arterial phase λHu of the energy curve is 3.71 HU/keV. **h**-**j** CT value of venous phase phase 40 keV, 70 keV and 100 keV monochromatic image is 299.1 HU, 118.4HU, 79.4HU, respectively. **k** Photomicrograph confirmed the pathological finding of the nodule as papillary thyroid carcinoma.(Hematoxylin-eosin stain; original magnification, 40.) **l** Venous phase NIC is 0.68. **m** Venous phase NZeff is 0.93. **n** Venous phase λHu of the energy curve is 3.66 HU/keV. AP arterial phase, VP venous phase, HU CT value, λHU the slope of spectral HU curve, NIC normalized iodine concentration, NZeff normalized effective atomic number, H&E Haematoxylin-eosin

**Fig. 7:**
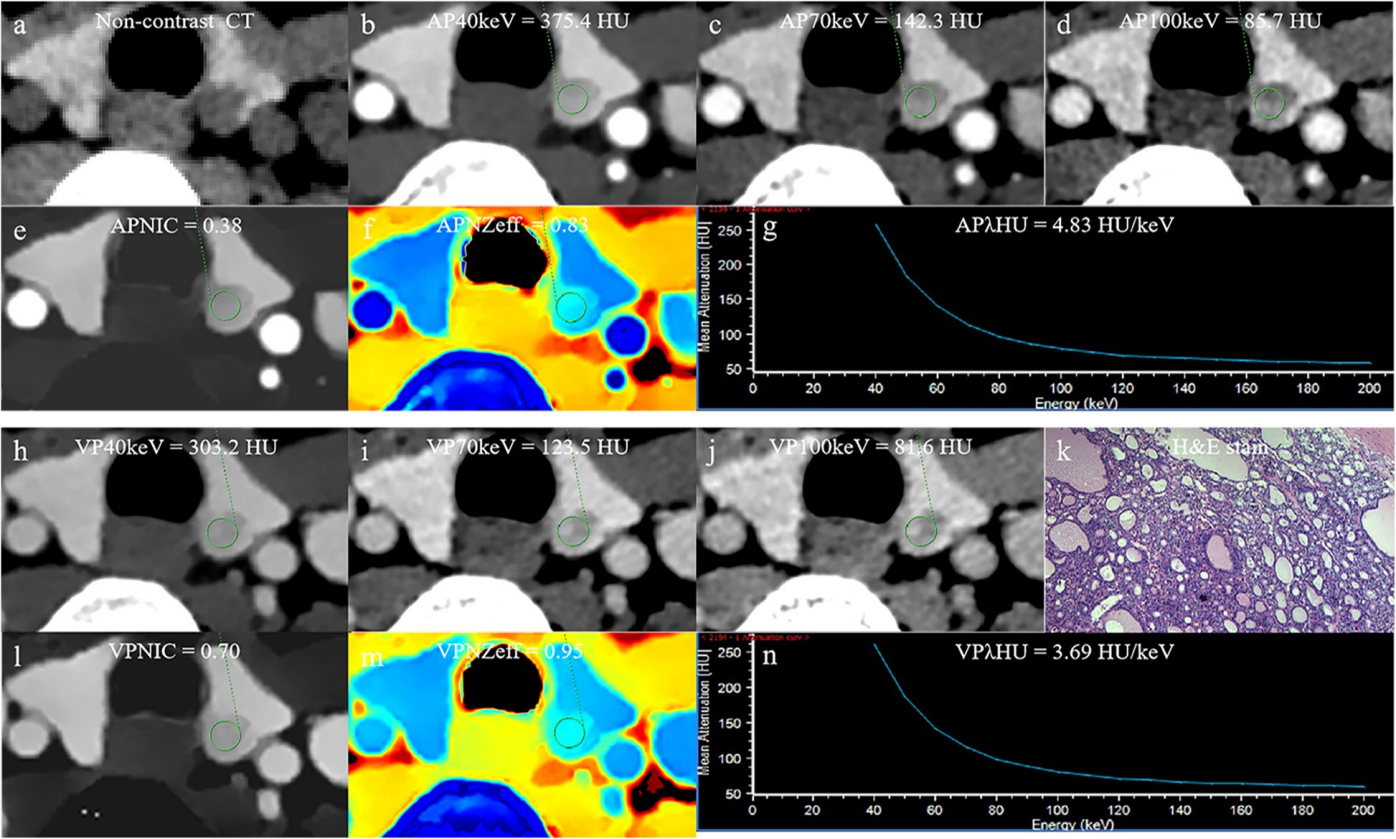
The DSCT quantitative parameters and Haematoxylin-eosin stain in a 38-year-old women with thyroid nodular goiters. **a** Axial non-contrast CT image shows the micro-nodule of thyroid left lobe. **b**-**d** CT value of arterial phase 40 keV, 70 keV and 100 keV monochromatic image is 375.4 HU, 142.3 HU, 85.7 HU, respectively. **e** Arterial phase NIC is 0.38. **f** Arterial phase NZeff is 0.83. **g** Arterial phase λHu of the energy curve is 4.83 HU/keV. **h**-**j** CT value of venous phase phase 40 keV, 70 keV and 100 keV monochromatic image is 302.2 HU, 123.5 HU, 81.6 HU, respectively. **k** Photomicrograph confirmed the pathological finding of the nodule as thyroid nodular goiters.(Hematoxylin-eosin stain; original magnification, 40). **l** Venous phase NIC is 0.70. **m** Venous phase NZeff is 0.95. **n** Venous phase λHu of the energy curve is 3.69 HU/keV. AP arterial phase, VP venous phase, HU CT value, λHU the slope of spectral HU curve, NIC normalized iodine concentration, NZeff normalized effective atomic number, H&E Haematoxylin-eosin

The DSCT-Radiological nomogram was constructed through a combination of the selected DSCT parameters and the radiologic features multiplied by their respective weighted coefficients. The formula was as follows: DSCT-Radiological nomogram = 5.478 + 0.892*micro-calcification+ 0.968*enhanced blurring-1.539*APλHU. The DSCT-Radiological nomogram for the identification of TMC and benign micro-nodules was established according to the APλHU+Radiological model (Fig. 3). The calibration curve of the nomogram demonstrated that the prediction result was in good agreement with the actual observation (Fig. 4). The DCA curve revealed that the nomogram can result in more net benefit than the all-or-none intervention strategy for all threshold probabilities (Fig. 5). The DSCT and pathological images from the 2 examples are shown in Figs. 6 and 7.

## Discussion

In this study, a nomogram combining quantitative DSCT parameters and conventional typical CT features for the identification of TMC and benign micro-nodule was established and showed good evaluation performance. This finding suggested that DSCT quantitative parameters can be a useful supplement to conventional CT radiological features for the differential diagnosis of TMC and benign micro-nodule. In addition, the DSCT-Radiological nomogram is easy to use with only relying on simple experience in the identification of typical radiological features and combining the quantitative parameter λHU, which is suitable for inexperienced first-line clinicians.

In our study, the quantitative parameters of DSCT including APλHU, APNIC, APNZeff, VPNIC and VPNZeff were able to independently identify TMC patients. After these quantitative parameters were analysed by ROC curves, APλHU demonstrated the highest efficacy with an AUC value of 0.829. These results of quantitative parameters in the current study are consistent with those in previous studies [16, 17], which can be used to diagnose thyroid lesions with good diagnostic efficiency. Our findings also revealed that the APNIC value of TMC was lower than that of benign micro-nodule. This may be because the tumour cells of thyroid malignant nodules destroy the thyroid follicular cells, which leads to a decrease in iodine uptake function and causes a reduction in the X-ray attenuation coefficient of the lesion [18]. In addition, a difference was observed in the quantitative parameter APλHU between TMC and benign micro-nodule because of the different nature of the nodules, the different number of thyroid follicular cells, and the variation patterns of iodine content. In the present study, the APλHU of malignant micro-nodule was found to be significantly lower than that of benign micro-nodule, which indicates that spectral curve analysis can help reveal the characteristics of lesions so that benign micro-nodule and TMCs may be differentiated, consistent with the findings from experiments reported by Jiang L et al. [19].

Our study also found that two classical radiological features, micro-calcification and enhanced blurring, could distinguish TMC and benign micro-nodule. Several previous studies have shown that the pathological basis of micro-calcification is the formation of a psammoma body in thyroid cancer and confirmed that this sign may be one of the independent risk factors for thyroid cancer [20–22]. Consistent with the above study, we also found a higher incidence of micro-calcification in the TMC group than in the benign micro-nodule group. In addition, our study results suggest that enhanced blurring could serve as a predictor of TMC. To our knowledge, this may be because the neovascularization in the TMC area adjacent to the thyroid parenchyma is more substantial than in the central region, which leads to a centripetal enhancement pattern of nodules and less density difference between tumour and normal thyroid tissue after enhancement [23, 24].

The typical conventional CT radiological features were added to the quantitative DSCT parameters model for further study, while satisfactory results for the identification of TMC and benign micro-nodules were obtained with multiple quantitative parameters of DSCT. After the diagnostic efficacy of these models that combined the quantitative DSCT parameters with typical radiological features was sequentially compared, it was found that the DSCT-Radiological nomogram, constructed with the quantitative DSCT parameter APλHU and the typical radiological features of micro-calcification and enhanced blurring, had the highest efficacy in identifying TMC and benign micro-nodule. The AUC of this DSCT-Radiological nomogram was 0.858, the sensitivity was 0.791, and the specificity was 0.800, which were higher than the corresponding values of other DSCT combined models. And this favorable performance might be similar to qualitative analysis by human experts or analysis by US [24]. However, it can be a bit difficult for inexperienced radiologists and US doctors to differentiate benign and malignant micro-nodules through qualitative analysis. Therefore, our proposed nomogram that the combination of quantitative DSCT parameters with typical radiological features may be a promising method to help improve the ability of first-line clinicians to distinguish TMCs and benign micro-nodules and make personalized clinical decision. In addition, this nomogram could visually estimate the nature of the micro-nodule. With the help of this DSCT-Radiological nomogram, individualized risk assessment in terms of predicting TMC nodules can be implemented for patients. Finally, this nomogram showed good agreement with the actual pathological results, such as the estimated benign and malignant risk outcomes, as seen in the calibration curves. Additionally, this DSCT-Radiological nomogram would result in a net benefit in the prediction of benign and malignant risk outcomes with almost all the ranges of threshold probabilities, and it may serve as a simple, potentially reliable and reproducible tool to guide clinical personalized assessment.

However, this study also had several limitations. First, this is a single-centre retrospective study with a relatively small sample size. In addition, all nodules included in this study had a maximum diameter of less than 1 cm, and thus, the DSCT-Radiological nomogram was not applicable to patients with large thyroid nodules. Second, since most of the malignant nodules in our study population were papillary thyroid microcarcinoma, the efficacy of our nomogram may be limited in the identification of other pathological types of malignant nodules. Third, this study only involves a single comparison of different quantitative parameters between the two groups, and did not evaluate the correlation between these DSCT quantitative parameters. Additionally, an external validation approach of the DSCT-Radiological nomogram may be beneficial to determine generalizability of the final model in the future.

## Conclusion

In conclusion, this study provides a DSCT-Radiological nomogram, consisting of the quantitative parameter APλHU derived from DSCT images and typical radiological features, which shows favourable performance and personalized risk assessment of identifying TMC patients with thyroid micro-nodules. This valid nomogram can assist first-line clinicians in identifying TMC patients and making personalized clinical decisions.

## Supplementary Information

**Additional file 1: Table S1.** Inter-reader reproducibility for measurements of DSCT parameters and radiological features. **Table S2.***P*-values of DeLong test for AUC of 8 different combinations. **Table S3.***P*-values of DeLong test for AUC of 7 different combinations.

## Data Availability

The datasets generated and/or analyzed during the current study are available from the corresponding author upon reasonable request.

## Abbreviations

DSCT: Double-layer spectral computed tomography
TMC: Thyroid microcarcinoma
ROC: Receiver operating characteristic
LR: Logistic regression
AUC: Area under the receiver operating characteristic curve
US: Ultrasound
FNAB: Fine-needle aspiration biopsy
IC: Iodine concentration
Zeff: Effective atomic number
ROI: Region of interest
AP: Arterial phase
VP: Venous phase
λHU: Slope of spectral HU curve
95% CIs: 95% confidence intervals
DCA: Decision curve analysis
