# Supplementary material for: Nomogram based on spectral CT quantitative parameters and typical radiological features for distinguishing benign from malignant thyroid micro-nodules
Source: Cancer Imaging. 2023 Jan 26;23:13. doi: 10.1186/s40644-023-00525-2 (PMC9878766; doi:10.1186/s40644-023-00525-2)
Supplement: Supplementary file 1 — Additional file 1: Table S1. Inter-reader reproducibility for measurements of DSCT parameters and radiological features. Table S2.P-values of DeLong test for AUC of 8 different combinations. Table S3.P-values of DeLong test for AUC of 7 different combinations. [file 40644_2023_525_MOESM1_ESM.docx]

**Table S1** Inter-reader reproducibility for measurements of DSCT parameters and radiological features

| Variable | ICC | 95% CI |
| --- | --- | --- |
| **AP** | | |
| 40keV(HU) | 0.963 | 0.954-0.970 |
| 70keV(HU) | 0.977 | 0.972-0.982 |
| 100keV(HU) | 0.955 | 0.945-0.964 |
| λHU | 0.964 | 0.956-0.971 |
| NIC | 0.883 | 0.858-0.905 |
| NZeff | 0.868 | 0.839-0.892 |
| **VP** | | |
| 40keV(HU) | 0.906 | 0.885-0.923 |
| 70keV(HU) | 0.916 | 0.897-0.932 |
| 100keV(HU) | 0.932 | 0.917-0.945 |
| λHU | 0.909 | 0.888-0.926 |
| NIC | 0.892 | 0.868-0.912 |
| NZeff | 0.871 | 0.843-0.895 |
| **Radiological features** | | |
| micro-calcification | 0.933 | 0.917-0.945 |
| enhanced blurring | 0.924 | 0.907-0.938 |

*AP* arterial phase, *VP* venous phase, *λHU* the slope of spectral HU curve, *NIC* normalized iodine concentration, *NZeff* normalized effective atomic number.

**Table S2** P-values of DeLong test for AUC of 8 different combinations.

|  | AP_40keV_+Radiological | AP_70keV_+Radiological | AP_100keV_+Radiological | APλHU+Radiological | APNIC+Radiological | APNZeff+Radiological | VPNIC+Radiological | VPNZeff+Radiological |
| --- | --- | --- | --- | --- | --- | --- | --- | --- |
| AP_40keV_+Radiological (AUC=0.857) | - | 0.0036^*^ | <0.0001^*^ | 0.8787 | 0.0153^*^ | <0.0001^*^ | <0.0001^*^ | <0.0001^*^ |
| AP_70keV_+Radiological (AUC=0.823) | 0.0036^*^ | - | <0.0001^*^ | 0.0405^*^ | 0.6843 | 0.0161^*^ | <0.0001^*^ | <0.0001^*^ |
| AP_100keV_+Radiological (AUC=0.721) | <0.0001^*^ | <0.0001^*^ | - | <0.0001^*^ | 0.0007^*^ | 0.1529 | 0.2208 | 0.1802 |
| APλHU+Radiological (AUC=0.858) | 0.8787 | 0.0405^*^ | <0.0001^*^ | - | 0.0148^*^ | 0.0001^*^ | <0.0001^*^ | <0.0001^*^ |
| APNIC+Radiological (AUC=0.813) | 0.0153^*^ | 0.6843 | 0.0007^*^ | 0.0148^*^ | - | <0.0001^*^ | 0.0001^*^ | 0.0001^*^ |
| APNZeff+Radiological (AUC=0.759) | <0.0001^*^ | 0.0161^*^ | 0.1529 | <0.0001^*^ | <0.0001^*^ | - | 0.0245^*^ | 0.0179^*^ |
| VPNIC+Radiological (AUC=0.693) | <0.0001^*^ | <0.0001^*^ | 0.2208 | <0.0001^*^ | 0.0001^*^ | 0.0245^*^ | - | 0.7067 |
| VPNZeff+Radiological (AUC=0.691) | <0.0001^*^ | <0.0001^*^ | 0.1802 | <0.0001^*^ | 0.0001^*^ | 0.0179^*^ | 0.7067 | - |

**P*-alue<0.05, *AP* arterial phase, *VP* venous phase, *λHU* the slope of spectral HU curve, *NIC* normalized iodine concentration, *NZeff* normalized effective atomic number.

**Table S3** P-values of DeLong test for AUC of 7 different combinations.

|  | enhanced blurring | micro-calcification | APλHU | micro-calcification+enhanced blurring | APλHU+micro-calcification | APλHU+enhanced blurring | APλHU+Radiological |
| --- | --- | --- | --- | --- | --- | --- | --- |
| enhanced blurring  (AUC=0.600) | - | 0.4957 | <0.0001^*^ | 0.0079 | <0.0001^*^ | <0.0001^*^ | <0.0001^*^ |
| micro-calcification  (AUC=0.623) | 0.4957 | - | <0.0001^*^ | 0.0013^*^ | <0.0001^*^ | <0.0001^*^ | <0.0001^*^ |
| APλHU (AUC=0.829) | <0.0001^*^ | <0.0001^*^ | - | <0.0001^*^ | 0.1449 | 0.0574 | 0.0172^*^ |
| micro-calcification+enhanced blurring(AUC=0.668) | 0.0079 | 0.0013^*^ | <0.0001^*^ | - | <0.0001^*^ | <0.0001^*^ | <0.0001^*^ |
| APλHU+micro-calcification  (AUC=0.842) | <0.0001^*^ | <0.0001^*^ | 0.1449 | <0.0001^*^ | - | <0.0001^*^ | 0.0474^*^ |
| APλHU+enhanced blurring (AUC=0.846) | <0.0001^*^ | <0.0001^*^ | 0.0574 | <0.0001^*^ | <0.0001^*^ | - | 0.1266 |
| APλHU+Radiological (AUC=0.858) | <0.0001^*^ | <0.0001^*^ | 0.0172^*^ | <0.0001^*^ | 0.0474^*^ | 0.1266 | - |

**P*-alue<0.05, *AP* arterial phase, *VP* venous phase, *λHU* the slope of spectral HU curve.
